# Innovative screening for OSAS in patients with head and neck cancer using the apneal application: Protocol for a prospective single-centre study

**DOI:** 10.1371/journal.pone.0346607

**Published:** 2026-06-23

**Authors:** Lisa Baluta, Maxime Humbert, Emmanuel Babin, Rémy Morello, Emmanuel Micault

**Affiliations:** 1 Service d’ORL et chirurgie cervico-faciale, CHU Caen Normandie, Caen, France; 2 Service d’ORL et chirurgie cervico-faciale, CHU Caen Normandie, Caen, France; 3 Service d’ORL et chirurgie cervico-faciale, CHU Caen Normandie, Caen, France; 4 Service de Biostatistique, CHU Caen Normandie, Caen, France; 5 Service d’ORL et chirurgie cervico-faciale, CHU Caen Normandie, Caen, France; Charité - Universitätsmedizin Berlin, GERMANY

## Abstract

**Introduction:**

OSAS is a common yet underdiagnosed condition, particularly among patients with head and neck cancers (HNC). Anatomical changes caused by the tumor or its treatments—surgery, radiotherapy, or chemotherapy—can promote the onset of OSAS. However, fatigue in these patients is often attributed solely to the cancer treatment itself, which may delay appropriate diagnosis and management.

**Methods:**

This prospective single-center study aims to assess the clinical feasibility of OSAS screening in patients with HNC using an innovative mobile application, *Apneal*, which uses smartphone sensors to detect nocturnal respiratory events through an artificial intelligence algorithm. Fifty adult patients diagnosed with HNC will be included. Each patient will perform two home-based overnight recordings using the *Apneal* application: one at the time of diagnosis and another six months after local treatment. Clinical and quality-of-life questionnaires will be completed. The estimated AHI will be compared with symptomatology and, if necessary, confirmed by a standard respiratory polygraphy.

**Expected Results:**

The primary outcome is the proportion of patients for whom *Apneal* data are usable at the 6-month follow-up after treatment. Secondary objectives include assessing the incidence of OSAS according to the oncologic treatments received, identifying high-risk profiles, and evaluating the impact of OSAS on quality of life.

**Discussion:**

This study could pave the way for a systematic, simple, low-cost, and non-invasive OSAS screening in patients with HNC. Ultimately, the goal is to enable a more personalized and comprehensive approach to care in this vulnerable population.

## Introduction

Obstructive Sleep Apnea Syndrome (OSAS) is a common condition in France, affecting approximately 4–10% of the population, but it remains underdiagnosed [1]. This syndrome is defined by episodes of obstructive apnea and/or hypopnea accompanied by daytime sleepiness. It can lead to fatigue, cardiovascular complications, motor vehicle accidents, and depressive or anxiety disorders [1]. The pathophysiology of OSAS is primarily based on a narrowing of the pharyngolaryngeal airway during sleep and/or a loss of function in the pharyngeal dilator muscles [2].

Head and neck cancers (HNC) account for approximately 15,000 new cases per year in France in 2018 according INCa (Institut National du Cancer) [3]. These cancers and their treatments (radiotherapy, chemotherapy, surgery with or without reconstruction) cause significant anatomical changes in the upper airway. Given that the main characteristic of OSAS is upper airway collapse, additional factors may further exacerbate this obstruction in this group of patients. These include anatomical abnormalities (such as hypertrophied soft tissues or narrowing of the pharyngeal space due to a mass effect) and neurological factors (such as neurosensory dysfunction affecting the feedback loop between the upper airways and the pharyngeal dilator muscles).

Therefore, it is reasonable to assume that patients with HNC may be at increased risk of developing OSAS. An increasing number of studies support this association, although the specific risk factors have not yet been clearly identified [4]. However, this condition appears to be underdiagnosed in this group of patients, as the chronic fatigue experienced by this population seems to be attributed to the cancer itself and its various treatments. Moreover, the distinction between sleepiness, the predominant symptom of obstructive sleep apnea (OSA), and asthenia is not always clearly made by either practitioners or patients.

Given these findings, it seems relevant to explore the implementation of OSAS screening in patients newly diagnosed with HNC and during follow-up. The aim of this work is to enable earlier intervention and improve the overall quality of life of these patients during their cancer care. Currently, fatigue in patients treated for HNC is often attributed to cancer treatments, and the contribution of a coexisting condition such as OSAS is rarely considered.

Access to polysomnography or ventilatory polygraphy remains difficult and costly for healthcare systems. For HNC patients, whose follow-up is already complex, a rapid diagnosis of OSAS would be particularly beneficial. Recently, new diagnostic tools have been developed for easy home use, based on tracheal sounds [5], mandibular movements [6], or motion-sensing mattresses [7]. However, these devices require dedicated equipment.

Our project aims to simplify the diagnosis of OSAS and reduce its cost. In this context, we have partnered with a start-up that has developed a smartphone-based medical application called APNEAL [8].

APNEAL is currently undergoing validation in a European clinical trial. It has already been tested in a preliminary French clinical study (Hôpital Bichat, AP-HP) to confirm its feasibility [8]. The application uses smartphone sensors, worn on the chest overnight, to detect apneas and hypopneas using an AI-based algorithm. In the first study, APNEAL’s results were compared to simultaneous polysomnography recordings and showed promising accuracy. In this first study conducted at Bichat Hospital in patients with a high probability of having obstructive sleep apnea syndrome (OSAS), it was shown that respiratory events could be accurately detected using smartphone sensors such as the microphone, gyroscope, and accelerometer. The results were compared with non-expert scorers analyzing polysomnography recordings and showed a sensitivity of 0.91 and a positive predictive value (PPV) of 0.89 for an apnea–hypopnea index (AHI) threshold of 15 [8].

The app is not yet commercially available.

Our study aims to assess the clinical feasibility of using this application in patients with HNC. The goal is to enable early detection of OSAS and, through repeated recordings, to identify patients who may be at higher risk of developing this condition.

## Materials and methods

This clinical study will be a prospective, monocentric, non-randomized trial conducted in a French department of Otorhinolaryngology and Head and Neck Surgery, expert in sleep disorder.

Inclusion criteria are as follows: adult patients affiliated with the national health insurance system, presenting with a suspected HNC, currently undergoing diagnostic work-up, and having signed an informed consent form. Eligible malignant tumor sites include the nasal cavities, sinuses, oral cavity, nasopharynx, oropharynx, hypopharynx, larynx, suspicious cervical lymphadenopathy and the parotid gland.Exclusion criteria include patients with a history of OSAS, prior treatment for HNC, minors, individuals under legal protection (guardianship or conservatorship), and later, patients whose lesion is not confirmed as malignant.

### Study population

Once included, each patient will follow an organized diagnostic pathway for suspected HNC. The study aims to enroll 2–3 patients per week over 6 months, totaling approximately 50 participants. If inclusion criteria are met, study participation will be proposed at the first consultation. Each patient will receive an information leaflet and provide written informed consent. Study logistics, potential risks, and procedures will be explained (V0).

### Study design

A second visit (V1) will include a standardized sleep consultation in accordance with the Association Française du Sommeil en ORL (AFSORL) [9], including the **Epworth Sleepiness Scale** [10] and **BOSS (Bordeaux Sleepiness Scale)** [11] for drivers. Demographic and clinical data will be collected (age, sex, comorbidities, treatments), along with several self-administered questionnaires:

- Snoring and sleep quality questionnaire (AFSORL) [9],
- EORTC QLQ-C30 (European Organisation for Research and Treatment of Cancer Quality Of Life) [12],
- EORTC H&N35 (European Organisation for Research and Treatment of Cancer Head and Neck) [13].

A loan smartphone (Samsung Galaxy XCover 7) with the APNEAL application pre-installed will be provided. Patients will conduct a home-based overnight recording under their usual sleeping conditions.

**Fig 1:**
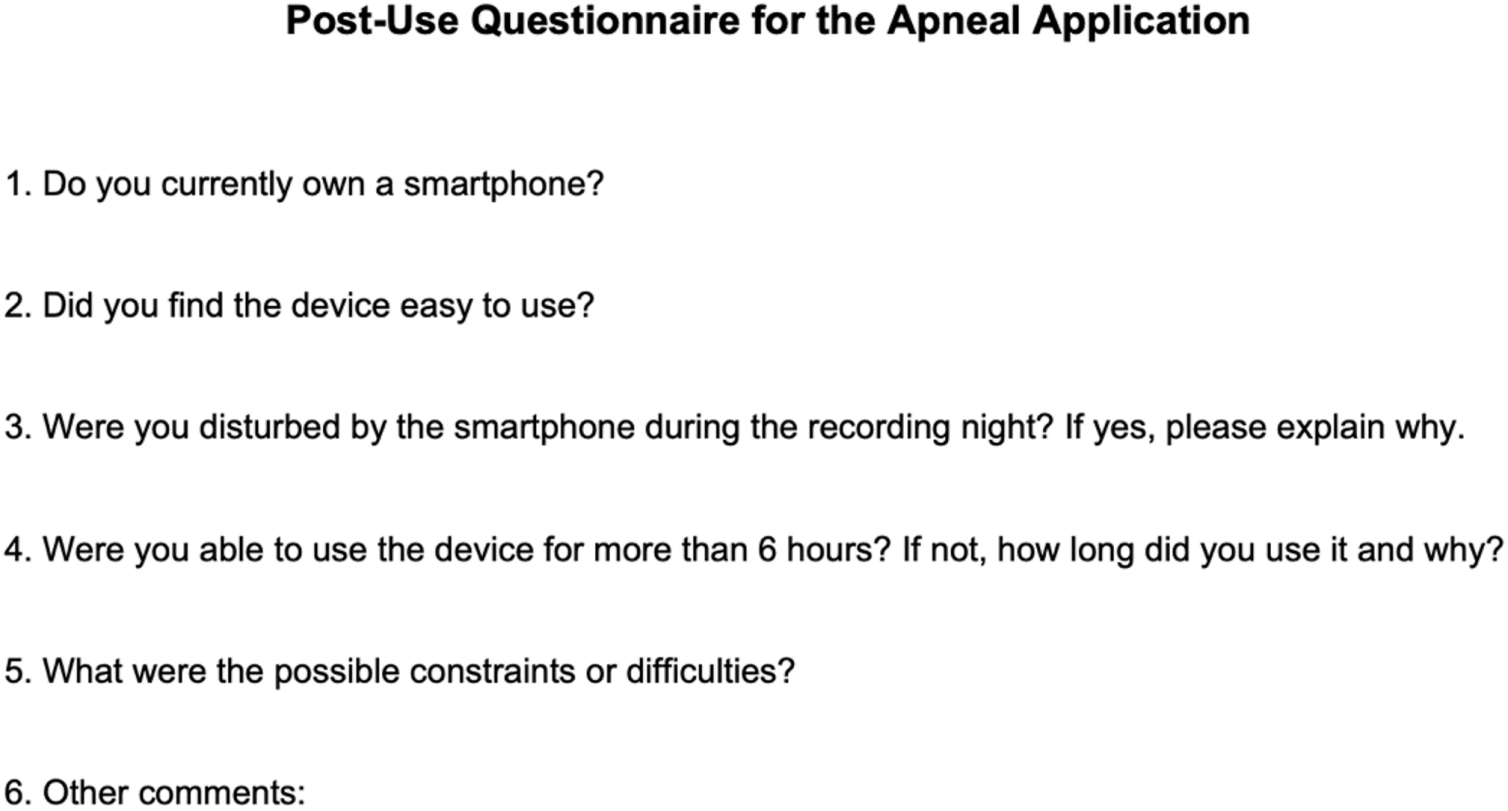
Post-use questionnaire for the APNEAL Application.

At a subsequent hospital visit (V2), the smartphone will be returned. Patients will complete a feedback questionnaire (Fig 1) on their experience with the application, and some will participate in semi-structured interviews.

Usable data will be extracted via a certified medical drive provided by MITRAL (developer of APNEAL), including recording duration and the estimated Apnea–Hypopnea Index (AHI).

**Fig 2:**
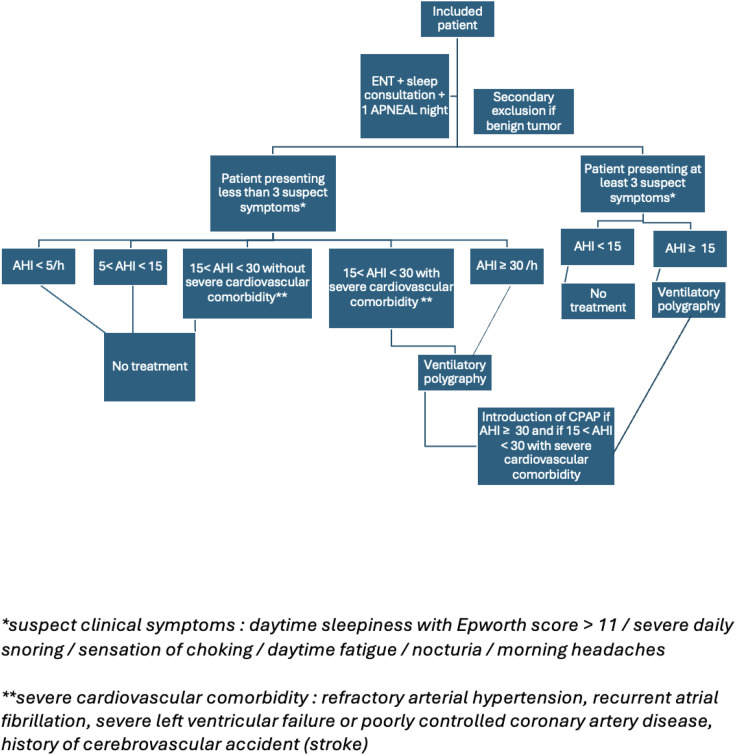
Flowchart for the management of the patient included according to symptomatology and the result given by the Apneal application.

Subsequent clinical management will be based on the AHI, symptomatology, and individual risk factors, following the flowchart (Fig 2). Given that the application is not yet officially validated at the start of the study, we have agreed that any AHI between 15 and 30 with severe cardiovascular comorbidity will require confirmation via a standard ventilatory polygraphy (*Cidelec*).

At the 6-month post-treatment follow-up visit (V3), in addition to the standard oncological follow-up consultation, patients will undergo a second sleep consultation integrated into their routine visit. This will include: a sleep consultation, the BOSS (Bordeaux Sleepiness Scale) questionnaire if the patient is a driver, and repeated completion of the self-administered questionnaires (Snoring and Sleep Quality Questionnaire per AFSORL, EORTC QLQ-C30, and H&N35). A second overnight home recording using the APNEAL application on the loan smartphone will also be performed. The V3 visit will take place 6 months after completion of the patient’s treatment (which is estimated to last approximately 3 months), except for metastatic patients receiving systemic therapy, who will be seen approximately 9 months after initiation of treatment.

**Fig 3:**
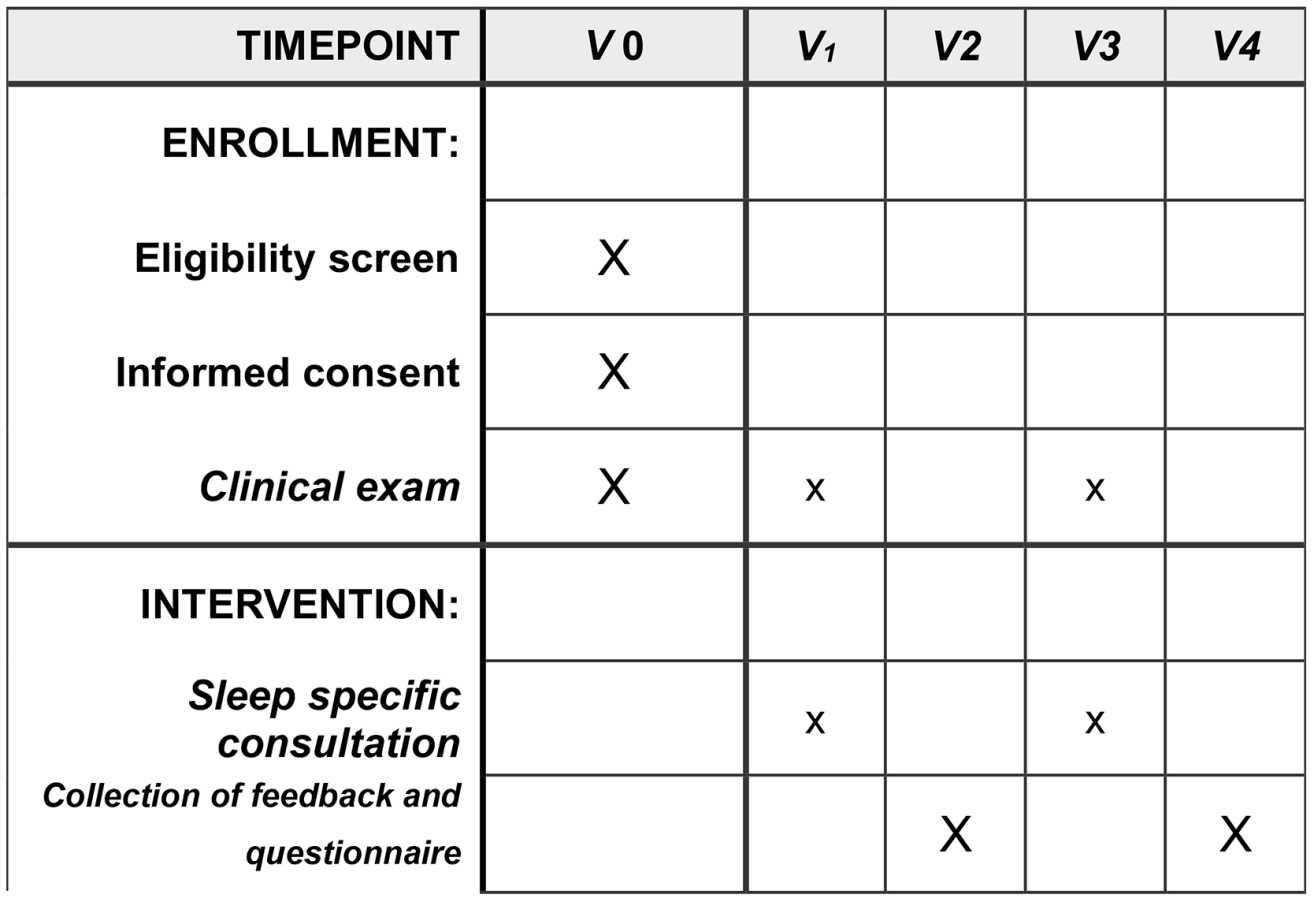
Participant timeline.

During a subsequent visit (V4), the patient will return the smartphone and complete questionnaires regarding their user experience. Clinical management will again follow the predefined flowchart (Fig 3).

The study has not yet started. Patient enrollment is scheduled to begin in October 2025, recruitment will be completed by May 2026, and patient follow-up is expected to finish around February 2027. The results are anticipated by May 2027.

### APNEAL: Instructions for use

**Fig 4:**
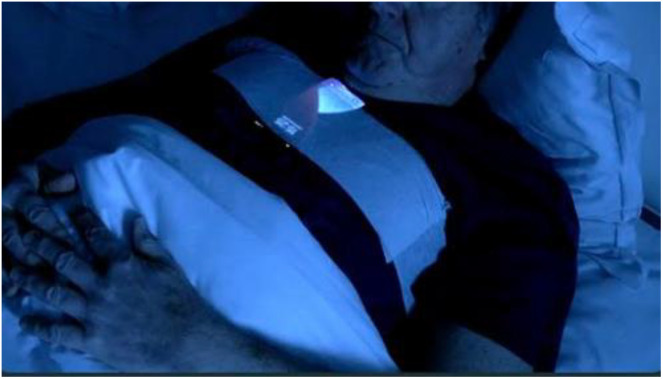
Photo showing how the smartphone should be attached [14].

The smartphone will be attached to the patient’s chest using adhesive tape, with the screen facing outward (Fig 3). To avoid burns from overheating and potential skin injuries from the adhesive, it is recommended to secure the phone over a comfortable piece of nightwear. The smartphone must be set to “airplane mode” to prevent the emission of electromagnetic waves. We will place the smartphone ourselves to ensure it is correctly positioned for the study. Using artificial intelligence algorithms, APNEAL analyzes the patient’s position during the night, the probability of breathing, and the probability of snoring in order to generate interpretable signals Fig 4.

### Judgement criteria

The primary endpoint of the study is to evaluate the feasibility of screening for obstructive sleep apnea syndrome (OSAS) in clinical practice among patients with head and neck cancers, using the APNEAL application.

The secondary endpoints are to examine the incidence of OSAS in patients treated for HNC depending on the therapeutic strategy used, in order to identify patient profiles at higher risk of developing OSAS, and to assess the impact on their quality of life.

To achieve this, the primary evaluation criterion is the proportion of patients with usable data collected 6 months after the end of local oncological treatment, with an interim analysis following the first consultation and the first overnight recording.

The secondary evaluation criteria include measuring the evolution of the apnea-hypopnea index (AHI) during cancer management to identify risk factors, and assessing the changes in quality of life indicators throughout the treatment process.

### Statistical analysis

The planned number of patients is 30. However, considering the risk of some patients not completing the study, it is entirely feasible to recruit up to 50 patients. For a 60% success rate of patients with usable collected data (i.e., 30 out of 50 patients), the 95% confidence interval is between 45.2% and 73.6%.

For the primary evaluation criterion, the proportion of patients with usable collected data will be calculated along with its exact 95% confidence interval. Regarding the incidence of OSAS, the first secondary objective, analysis will focus on OSAS classification (mild, moderate to severe) according to the therapeutic strategy applied. To identify patients at higher risk of developing OSAS (second secondary objective), we will measure the AHI (Apnea-Hypopnea Index) throughout the care process. Changes in AHI scores, whether treated as quantitative or categorical data ([5–15]: mild OSAS; [15–30]: moderate OSAS; ≥ 30: severe OSAS), will be analyzed in relation to patients’ clinical characteristics and scale scores.

Statistical analyses will rely on standard univariate tests depending on the type of variables considered: Chi-square test, Fisher’s exact test, ANOVA, Pearson or Spearman correlation. Multivariate analyses will be considered depending on the analytical approach and the preliminary results (ANOVA, linear regression, logistic regression). A factor analysis using principal component analysis (PCA) may be performed to identify patient profiles, adhering to the validity conditions: the number of variables (quantitative or categorical with ≥3 categories) must correspond to at least 10× to 5 × the number of patients (i.e., 5–10 variables in the multifactorial model).

For the third secondary objective, which is to assess the impact on quality of life, changes in quality of life scores before and after treatment will be analyzed using a paired-sample test (Student’s paired t-test for V2 and V4, or the Wilcoxon signed-rank test).

All statistical analyses will be performed with a two-sided alpha risk of 5%, using IBM SPSS Statistics for Windows, Version 23.0. Armonk, NY: IBM Corp.

### Ethics

The protocol number is ID-RCB 2024-A02375-42, version no. 3 dated 02/27/2025, and it received a favorable opinion from the CPP Ouest I ethics committee on 03/06/2025. The study was approved by the ANSM on 02/21/2025. Recruitment is scheduled to begin in September 2025, with a 6-month inclusion period and an approximate follow-up of 10 months per patient. The study was registered on ClinicalTrials.gov under the identifier NCT06896448 on 03/27/2025.

This project was funded by CHU Caen Normandie through its internal INNOV’ ECLAIR call for projects.

## Discussion

Fatigue during oncological treatment is often attributed to the therapies the patient has undergone. However, an increasing number of studies are emerging, highlighting a growing association between the presence of a malignant tumor in the upper aerodigestive tract and the onset of obstructive sleep apnea syndrome (OSAS) [15], particularly following treatment [4], due in part to its impact on the pharyngeal dilator muscles.

Most studies published investigate the prevalence of OSAS post-treatment through retrospective analyses [16,17]. These generally reveal a higher prevalence in oropharyngeal cancers, which are more frequently studied [18,19]. This may be due to the oropharynx being a key anatomical junction of the upper airways, making it more susceptible to structural changes that can lead to OSAS. Nevertheless, other locations such as the hypopharynx and larynx also appear to present significant risk, though they are less extensively studied. In several studies, no significant differences were found between treatment modalities, such as surgery versus chemoradiotherapy [19]. However, others suggest surgery—particularly when involving free flap reconstruction [19]—may be associated with a higher risk of OSAS [20]. Moreover, intermittent hypoxia has been shown to promote the progression of HNC, potentially compromising treatment outcome [16].

Therefore, we believe it is relevant to explore this topic through a prospective study, in order to monitor the evolution of OSAS from the start to the end of the care pathway. Importantly, this approach may help identify high-risk patient profiles who could benefit from **systematic, easy-to-use screening**—applicable to all types of HNC.
